# *Anaplasma phagocytophilum* and *Anaplasma ovis*–Emerging Pathogens in the German Sheep Population

**DOI:** 10.3390/pathogens10101298

**Published:** 2021-10-09

**Authors:** Benjamin Ulrich Bauer, Cristian Răileanu, Oliver Tauchmann, Susanne Fischer, Christina Ambros, Cornelia Silaghi, Martin Ganter

**Affiliations:** 1 Clinic for Swine and Small Ruminants, Forensic Medicine and Ambulatory Service, University of Veterinary Medicine Hannover, Foundation, Bischofsholer Damm 15, 30173 Hannover, Germany; martin.ganter@tiho-hannover.de; 2 Institute of Infectiology, Friedrich-Loeffler-Institut, Suedufer 10, 17493 Greifswald Isle of Riems, Germany; cristian.raileanu@fli.de (C.R.); oliver.tauchmann@fli.de (O.T.); susanne.fischer@fli.de (S.F.); cornelia.silaghi@fli.de (C.S.); 3 Sheep Health Service, Bavarian Animal Health Service, Senator-Gerauer-Straße 23, 85586 Poing-Grub, Germany; christina.ambros@tgd-bayern.de; 4 Faculty of Mathematics and Natural Sciences, University of Greifswald, Domstraße 11, 17489 Greifswald, Germany

**Keywords:** *Dermacentor marginatus*, emerging diseases, *Ixodes ricinus*, One Health, ovine anaplasmosis, sheep, tick-borne fever

## Abstract

Knowledge on the occurrence of pathogenic tick-borne bacteria *Anaplasma phagocytophilum* and *Anaplasma ovis* is scarce in sheep from Germany. In 2020, owners from five flocks reported ill thrift lambs and ewes with tick infestation. Out of 67 affected sheep, 55 animals were clinically examined and hematological values, blood chemistry and fecal examinations were performed to investigate the underlying disease causes. Serological tests (cELISA, IFAT) and qPCR were applied to all affected sheep to rule out *A. phagocytophilum* and *A. ovis* as a differential diagnosis. Ticks were collected from selected pastures and tested by qPCR. Most animals (*n* = 43) suffered from selenium deficiency and endoparasites were detected in each flock. *Anaplasma* spp. antibodies were determined in 59% of examined sheep. Seventeen animals tested positive for *A. phagocytophilum* by qPCR from all flocks and *A. phagocytophilum* was also detected in eight pools of *Ixodes ricinus*. *Anaplasma phagocytophilum* isolates from sheep and ticks were genotyped using three genes (*16S rRNA*, msp4 and groEL). *Anaplasma ovis* DNA was identified in six animals from one flock. Clinical, hematological and biochemical changes were not significantly associated with *Anaplasma* spp. infection. The *16S rRNA* analysis revealed known variants of *A. phagocytophilum*, whereas the msp4 and groEL showed new genotypes. Further investigations are necessary to evaluate the dissemination and health impact of both pathogens in the German sheep population particularly in case of comorbidities.

## 1. Introduction

*Anaplasma phagocytophilum* and *Anaplasma ovis* are tick-borne obligate intracellular bacteria. Wild ungulates are considered to be reservoirs for both *Anaplasma* species [1,2,3,4].

In Europe, *A. phagocytophilum* is mainly transmitted by the tick species *Ixodes ricinus* [5,6]. The pathogen replicates within vacuoles in neutrophilic granulocytes and causes granulocytic anaplasmosis in multiple species such as horses, dogs, cats and tick-borne fever (TBF) in ruminants [7,8]. Besides animals, *A. phagocytophilum* causes human granulocytic anaplasmosis (HGA), which is widely distributed across Europe and the USA [6]. In sheep, TBF mainly affects lambs and most characteristic signs are high fever, anorexia, dullness, nasal discharge, lacrimal secretion and in some cases TBF is fatal [9,10]. Some infected lambs have a reduced weight gain (ill thriftiness) [11,12]. Neutropenia and thrombocytopenia are the hematological key findings in *A. phagocytophilum* infected sheep [13,14]. As a consequence of the induced immunosuppression, secondary infections, mainly with *Mannheimia haemolytica* and *Bibersteinia trehalosi,* occur and result in respiratory distress affecting the lamb’s health significantly [15,16]. Moreover, co-infection with staphylococcal bacteria in lambs can result in tick pyemia with severe polyarthritis [17].

By contrast, *A. ovis* causes ovine anaplasmosis and seems to be more host-specific than *A. phagocytophilum*. This pathogen mainly affects the ovine and caprine erythrocytes but can also be found in wild ungulates such as roe deer and red deer [2,18,19,20]. Infections with *A. ovis* in humans have hardly been reported so far [21]. *Anaplasma ovis* is widely distributed in the Mediterranean basin, appears rarely in Central Europe and has not yet been detected in Northern Europe [5,20,22,23]. The pathogen is considered to be transmitted by ticks of the genera *Dermacentor*, *Rhipicephalus*, and *Hyalomma* [24], but recent data about their vector competence are lacking. Lately, *A. ovis* was also found in sheep keds (*Melophagus ovinus)* [25,26]. The main clinical signs in sheep are severe anemia, extreme weakness, anorexia and weight loss, but these signs mainly occur under poor health conditions [18,27,28]. Hemoglobinuria was described in a sheep flock from Hungary [20] and icteric carcasses of *A. ovis* positive lambs were recently reported in Spain [27]. Hemolytic anemia is induced by *A. ovis* and results in a reduction in hematological parameters such as red blood cells (RBC), hemoglobin (Hb) and packed cell volume (PCV) in sheep experimentally infected with the pathogen [29].

There is a high diversity of *A. phagocytophilum* genetic strains circulating in animals and ticks. Molecular characterization of *A. phagocytophilum* relies mainly on the analyses of various loci such as *16S rRNA* locus, *groESL* operon, major surface protein coding genes (*msp2* and *msp4*) and *ankA* genes or on multilocus sequence typing approaches [30,31,32,33]. The distinction between pathogenic and apathogenic variants was previously proposed and it was possible to associate variants to different hosts [31,33,34,35,36]. In general, different strains of *A. phagocytophilum* circulating within a sheep flock have different pathogenic potential [9,37].

In Germany, the most common tick species is *I. ricinus* and the presence of *A. phagocytophilum* in *Ixodes* spp. and several domestic and wild animals was reviewed by Stuen and colleagues [7]. Ticks of the genus *Dermacentor* are more focally distributed in Germany, but the dissemination of *D. reticulatus* and *D. marginatus* has increased in recent years [38]. Information about the presence and genetic variants of *A. phagocytophilum* in German sheep flocks is scarce [3], while data about *A. ovis* in Germany is missing.

The first objective of the present study was to investigate the cause for ill thriftiness in lambs and ewes from five different sheep flocks in Germany. Hematological values, blood chemistry and fecal examinations were performed. Moreover, blood samples were examined for the presence of *A. phagocytophilum* and *A. ovis* by antibody detection, microscopical evaluation of blood smears, and qPCR. The role of *Anaplasma* spp. infection in clinical and clinicopathological abnormalities was evaluated. In addition, the study aimed to analyze the genotype diversity obtained from sheep blood, questing ticks and ticks collected from sheep.

## 2. Results

### 2.1. Clinical and Clinicopathological Findings

Out of all 55 sheep examined in July 2020, 42 sheep were emaciated or thin (BCS ≤ 2) and 22 animals showed nasal discharge. During examination, 11 sheep coughed/wheezed, and six lambs had lacrimal secretion. The conjunctiva of four lambs were pink-white (FAMACHA©Score 4). Three lambs had a marginal increase in body temperature (>40.5 °C).

Anemia and monocytopenia were the most frequent complete blood count (CBC) abnormalities and found in 49% and 40% of examined animals. Anemia was mild and moderate in 74% and 26% of cases, respectively. Among these anemic animals, most sheep had a normocytic hypochromic (56%) or microcytic hypochromic (30%) anemia. In addition, a few animals showed a normocytic normochromic (7%) or microcytic normochromic (7%) anemia.

Thrombocytopenia was detected in 34% of sheep and eosinophilia in 18%. None of the 55 sheep had neutropenia, but one ewe (T817.13) and four lambs suffered from neutrophilia.

The most frequent biochemical abnormality was hyperglobulinemia (96%). Hyperproteinemia and hypoalbuminemia was identified in 55% and 76% of the examined sheep, respectively. Hyperbilirubinemia was also diagnosed in 76% of sheep. Five lambs showed increased aspartate aminotransferase (AST) activities thereof one lamb (T820.2) had an elevated creatine kinase (CK) activity. Two lambs (T921.4, T921.6) had increased glutamate dehydrogenase (GDH) values. Creatinine concentrations were elevated in 11 lambs and decreased in one lamb (T921.5). The serum selenium levels were low (≤ 80 µg/L) in 78% of examined sheep including all animals from flock A, B and D.

Details of the clinical examination, blood and biochemical values are summarized in Supplementary Table S1.

### 2.2. Fecal Parasitological Examinations

In lambs, gastrointestinal nematode infection (mean ± standard error) was detected in flock A (Trichostrongylidae 100 ± 31.4 EpG), flock C (Trichostrongylidae 283 ± 52 EpG, *Nematodirus* spp. 89 ± 35.1 EpG) and flock D (Trichostrongylidae 985 ± 155.8 EpG, *Nematodirus* spp. 10 ± 6.7 EpG). Nematode eggs were not found in lambs from flock B and E. Two of the three ewes from flock A also had a Trichostrongylidae infestation (EpG 100 and 900). *Eimeria* spp. (mean ± standard error, percentage of *E. ovinoidalis*) were detected in each flock: flock A: 4388 ± 1829 OpG, 25.6%, flock B: 20,217 ± 6070 OpG, 5%, flock C: 4832 ± 2456 OpG, 55.6%, flock D: 3230 ± 807 OpG, 0% and flock E: 10,160 ± 3452 OpG, 24.5%.

Raw data about fecal examinations are presented in Supplementary Table S1.

### 2.3. Anaplasma phagocytophilum and Anaplasma ovis Laboratory Investigations

#### 2.3.1. Antibody Detection

Antibodies against *Anaplasma* spp. were detected in 38 sheep (*n* = 66), 28 animals were detected simultaneously by cELISA and indirect immunofluorescence antibody test (IFAT). Eight sheep tested seropositive solely by IFAT and three sheep were identified only by cELISA (Table 1).

#### 2.3.2. Detection by Microscopical Evaluation of Blood Smears

**Figure 1:**
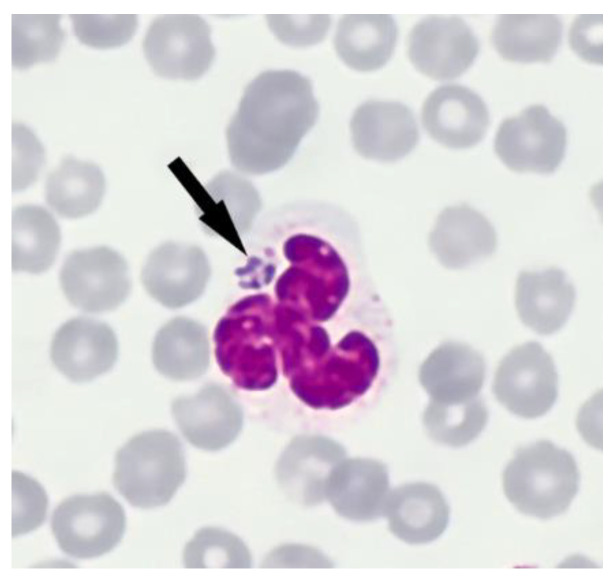
*Anaplasma phagocytophilum* morulae (arrow) within an infected neutrophil (May-Grünwald Giemsa stain ×1000) from lamb T819.10.

Intracytoplasmic morulae were identified in granulocytes of blood smears from two lambs (T819.10, T820.8) (Figure 1) and these lambs also tested *A. phagocytophilum* positive by qPCR. Inclusion bodies as morphological proof for *A. ovis* were not detected in blood smears.

#### 2.3.3. DNA Detection in Blood and in Ticks

Out of 67 examined sheep, 16 lambs and one ewe (25%) tested qPCR positive for *A. phagocytophilum* (Table 1). At least one lamb was qPCR positive in each flock. Twelve *A. phagocytophilum* PCR positive lambs from flocks A, B and D also tested positive with both serological tests. Four *A. phagocytophilum* DNA positive lambs were also positive by IFAT and one lamb (T921.2) tested positive solely by PCR (Table 1).

*Anaplasma ovis* DNA was detected in six animals only in flock A (32%): two lambs, three ewes and the breeding ram. Co-infection with *A. phagocytophilum* was identified in one ewe (T668.1). All *A. ovis* DNA positive sheep (except of the ram) tested serologically positive by ELISA and IFAT (Table 1).

Twelve *Anaplasma* DNA negative sheep tested simultaneously positive by cELISA and IFAT, whereas four sheep were only *A. phagocytophilum* positive by IFAT and three sheep were identified solely by cELISA.

On all three pastures near flock B, 256 questing *I. ricinus* ticks were collected (20 adults and 236 nymphs) in July 2020. The qPCR detected *A. phagocytophilum* DNA in 5% (1/20) of adult ticks, while the minimum infection rate in pooled nymphs was 3.4% (8/236). All tick samples tested negative for *A. ovis.*

The seven engorged ticks collected from sheep (flock A) in November 2020 included three *I. ricinus* females and four *D. marginatus* males. In the pool of *D. marginatus*, *A. ovis* was identified (Cq 24.2) but not *A. phagocytophilum*. The pool of *I. ricinus* tested negative for *Anaplasma* DNA.

##### *Anaplasma phagocytophilum* and *Anaplasma ovis* Sequencing

The nomenclature of the detected *A. phagocytophilum* variants is based on the nominations given by other authors [34,35,36,39]. The examined lambs showed altogether four *16S rRNA* gene variants: 16S-20(W) (*n* = 5), 16S-16(S) (*n* = 7), 16S-2(B) (*n* = 2), 16S-21(X) (*n* = 1) (Table 2).

The sequencing of the *16S rRNA* locus of *A. phagocytophilum* showed that both positive ticks found near flock B on one pasture had variant 16S-22(Y) and three positive ticks on the other pasture (flock B) showed variant 16S-21(X), whereas the sheep in flock B had shown variants 16S-16(S) and 16S-20(W) (Table 2 and Table 3).

For further analyses of diversity of *A. phagocytophilum msp4* and *groEL* loci were also sequenced. In total, valid sequences were produced for the *groEL* locus out of four *I. ricinus* and 14 sheep samples, and for *msp4* out of five *I. ricinus* and 14 sheep samples. For both loci so far, unknown variants could be also detected within the sheep and tick samples (Table 2 and Table 3).

**Figure 2:**
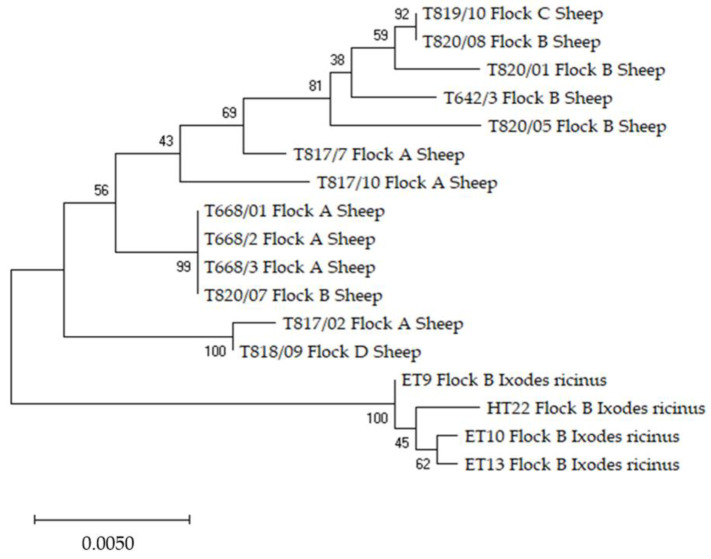
Phylogenetic clustering by concatenation of *16S rRNA*, *groEL* and *msp4* of *Anaplasma phagocytophilum* derived from sheep and *Ixodes ricinus*. The evolutionary analyses were conducted with MEGA X [40] using the Maximum Likelihood method and Tamura 3-parameter model [41]. Statistical support was calculated by 1000 bootstrap replicates, and the tree is scaled with branch lengths indicating the number of substitutions per site.

The phylogenetic analysis for each of the three individual loci indicated a cluster separation between the sequences of *A. phagocytophilum* from sheep to those from *I. ricinus* ticks (Figures S1–S3). The exception is represented by the *16S* tree in which the sheep isolate T921/02 from flock E, identified as variant 16S-21(X), clustered within the same clade with the isolates from ticks which are also 16S-21(X) variants. For all samples with three individual loci sequences, we combined these by concatenation, resulting in a larger sequence for every individual sample with higher discriminatory power than individual ones. The relationships among these samples can be seen in Figure 2. The concatenated tree confirmed the cluster separation of isolates from sheep to those from ticks observed after the phylogenetic analysis for each individual target.

The sequences of *A. phagocytophilum 16S rRNA*, *groEL* and *msp4* partial genes and *A. ovis msp4* from this study are available in GenBank under the accession numbers: MZ348247-MZ348306 and MZ363472-MZ363474. Table S3 contains information regarding the sequences from GenBank used for comparison and the obtained identities after BLAST analysis. All six *A. ovis* sequences showed 100% identity with several *A. ovis* isolates from GenBank (GenBank access. nos.: MT344082, LC229602 or MN198191).

### 2.4. Statistical Outcomes

None of the clinical findings, hematological variations and biochemical abnormalities were associated with either the detection of *Anaplasma* spp. DNA or the seropositivity to *Anaplasma* spp. (*p* > 0.05). Details are summarized in Supplementary Table S2.

## 3. Discussion

The present study investigated the cause for ill thriftiness in lambs and adult sheep. Besides selenium deficiency and endoparasitism, *A. phagocytophilum* was detected in animals from all five flocks. Additionally, *A. ovis* was discovered in one sheep flock for the first time in Germany. The hematological and biochemical abnormalities were not statistically related to the presence of *Anaplasma* spp. DNA or of antibodies against *Anaplasma* species. The analysis was hampered by the presence of selenium deficiency and endoparasitism, and both diseases influence hematological and biochemical values [42,43,44]. Nevertheless, it is still worth discussing the major findings. Neutropenia has been described as one of the key findings of *A. phagocytophilum* infected sheep, but this was not the case in the present study [13,45]. However, thrombocytopenia and monocytopenia were diagnosed in lambs from every flock. A reduced number of platelets were also frequently reported in horses, dogs and sheep clinically affected by *A. phagocytophilum* [14,46,47,48]. Moreover, monocytopenia was also found in cattle suffering from TBF [35]. Sheep which underwent an acute *A. phagocytophilum* infection had increased mean corpuscular hemoglobin (MCH) values [14]. This is in contrast to the findings of the present study. Around half of the examined lambs showed a mild to moderate anemia, which was mainly normocytic hypochromic or microcytic hypochromic and possibly caused by iron or copper deficiency [49,50,51]. Furthermore, secondary iron deficiency might result from chronic infections [51]. The hyperglobulinemia in almost all sheep is an indication of chronic inflammatory diseases such as pleuritis and pleural abscesses [50,52], but hyperglobulinemia was also found in dogs and horses with clinical granulocytic anaplasmosis [46,53]. Hyperproteinemia, hypoalbuminemia and hyperbilirubinemia have been reported from dogs with canine granulocytic anaplasmosis (CGA) [46]. Hyperbilirubinemia was diagnosed in 42 lambs in the present study and half of these animals had reduced hemoglobin values. This might be associated with selenium deficiency. Under experimental conditions, the selenoenzyme glutathione peroxidase protects hemoglobin against oxidation in murine and human erythrocytes, and low selenium levels might reduce the erythrocyte lifespan [54,55]. Another reason for hyperbilirubinemia may be the intake of toxic plants such as St. John’s wort (*Hypericum perforatum*), which is a common plant on extensive pastures in Germany [56,57,58]. In total, we assume that the examined sheep have already overcome the acute phase of an *Anaplasma* spp. infection or were only mildly infected with the pathogens. Additionally, all PCR positive animals tested antibody positive to at least one serological test, with the exception of one lamb in flock E. This means, that we did not have the chance to evaluate animals in an early phase of infection before seroconversion. In the end, the influence of *A. phagocytophilum* and *A. ovis* on the hematological and biochemical changes remains doubtful in the present study.

The applied cELISA has only been evaluated for *A. ovis* [59]. However, the cELISA is based on the use of ANAF16C1 Mab that recognizes the MSP5 antigen of all known *Anaplasma* spp. and is therefore suitable to diagnose also antibodies against *A. phagocytophilum* [60,61]. Cross-reactivity between *A. phagocytophilum* and *A. marginale* was described with the IFAT and this probably also occurs with *A. ovis* [62,63]. Therefore, species distinction based on serology is impossible with these methods. Nevertheless, the cELISA is a fast and inexpensive tool to screen large numbers of serum samples for *Anaplasma* spp. antibodies in flocks with an unknown status [60].

Not all seropositive sheep also tested positive by qPCR. *Anaplasma ovis* is detectable by qPCR for at least 300 days post infection [18]. In contrast, *A. phagocytophilum* circulates in undulation in sheep [64,65]. This feature of *A. phagocytophilum* might be responsible for the missing DNA detection in some seropositive animals.

The comparison of our results with findings from other studies is difficult due to the different study designs to identify *Anaplasma* spp. infections in small ruminants. In the present study, *A. phagocytophilum* DNA was detected in 25% of examined preselected sheep. In a previous study from Germany, 4% (*n* = 255) of the analyzed ovine samples tested *A. phagocytophilum* positive by PCR [3]. This discrepancy with our results might be due to the different sampling approaches. Ill thrifty sheep were examined in the present study while samples from a surveillance program were used by Scharf and colleagues [3]. In a Danish sheep flock, a quarter of unthrifty sheep were *A. phagocytophilum* PCR positive [66]. One of the highest infection rates was reported in lambs from Norway with 37.5% PCR positive animals [67]. On the contrary, a low *A. phagocytophilum* DNA detection of around 1% and 4% was identified in sheep from the Czech Republic and Slovakia, respectively [23]. In Italy, sheep in poor health conditions tested *A. phagocytophilum* DNA positive with an infection rate of 11.5% [28]. Finally, *A. phagocytophilum* circulates within sheep populations across Europe, but the extent of infection seems to vary among countries. These variations may be related to different husbandry systems, climate conditions and occurrence of *A. phagocytophilum* in the tick population.

*Anaplasma ovis* is endemic in many countries worldwide [22]. In Europe, this pathogen was mainly found in sheep and goats in Mediterranean countries, including one human case of *A. ovis* infection in Cyprus [20,21]. For instance, high detection rates of *A. ovis* DNA were reported in sheep (37%) from Italy (Sicily) and in goats (52%) from France (Corsica) [28,68]. In sheep flocks from Spain, 91.1% of the flocks tested PCR positive for *A. ovis* [69]. There are increasing reports about the presence of *A. ovis* in sheep and goats from countries located in Central Europe such as Hungary and Slovakia [20,23]. To the authors’ best knowledge, this is the first detection of *A. ovis* in Germany and the most northern occurrence of this pathogen in Central Europe at this time. The vector, *D. marginatus*, is present in the current study area [38], but *A. ovis* was identified only in one sheep flock located in the uplands of south Rhön, while the other four flocks grazed in the Spessart area. Therefore, *A. ovis* seems to occur only in a restricted area, but this needs further investigation by examining more sheep flocks and/or ticks in Lower Franconia. Moreover, *A. ovis* DNA was identified in an engorged *D. marginatus* collected from a sheep from flock A, but not in engorged *I. ricinus*. This detection does not prove the vector competence of *D. marginatus* due to the fact, that *A. ovis* might be acquired from infected sheep via a blood meal. In general, information about vectors of *A. ovis* in Europe is scarce, but the *Dermacentor* species are suspected to transmit the pathogen [20,24]. Additional research is essential to elucidate the role of *Dermacentor* spp. in the transmission of *A. ovis* due to the focal dissemination of *D. marginatus* and *D. reticulatus* across Germany [38].

A co-infection with both *A. phagocytophilum* and *A. ovis* was identified in one ewe (T668.1) by qPCR. This ill thrifty sheep had white-pink conjunctiva (FAMACHA©Score 4) according to the local veterinarian. Co-infections with both *Anaplasma* species were identified by PCR in 0.6% and 6.5% of examined sheep from Slovakia and Italy, respectively [23,28]. Unfortunately, adequate field clinical studies are lacking, and it is not clear whether simultaneous infection with both pathogens has more severe clinical consequences in small ruminants [22].

In flocks A and B, different *16S rRNA* gene variants of *A. phagocytophilum* were detected, which is in line with findings in Norwegian sheep flocks [37,67]. Variant ‘16S-20(W)’ was the most frequently detected variant (flock A, B, C), it corresponds to the prototype variant as the cause of TBF in ruminants [13,35]. This variant was previously also identified in other ruminants such as in mouflon (*Ovis musimon*), roe deer (*Capreolus capreolus*), fallow deer (*Dama dama*), red deer (*Cervus elaphus*), sika deer (*Cervus nippon*) and cattle in Germany [1,35,39,70,71].

Variant 16S-2-(B) has been described as the prototype variant of the HGA agent (GenBank access. no.: U02521). It has also been shown that sheep can carry a human *A. phagocyotphilum* isolate and can act successfully as hosts for infections of *Ixodes* ticks without showing any obvious clinical signs [61]. Although, the ‘B’ variant was identified in only two animals but from two different flocks (A, D), but not in ticks in the present study, *I. ricinus* can host the zoonotic variant [72,73]. This emphasizes the health risk for sheep farmers, forest workers, hunters, veterinarians and residents in the study area, and underlines the essential need of a One Health approach to prevent *Anaplasma* infections in animals and humans alike. Moreover, the ‘B’ variant was also detected in other species from Germany: in mouflon (*Ovis musimon*), fallow deer (*Dama dama*), sika deer (*Cervus nippon*), red deer (*Cervus elaphus*), and in horses and dogs with clinical granulocytic anaplasmosis [1,34,70,74].

The *16S rRNA* gene variant 16S-16-(S) found in lambs in flocks A and B has been previously detected in horses with clinical equine granulocytic anaplasmosis, and also in mouflon (*Ovis musimon*), sika deer (*Cervus nippon*), fallow deer (*Dama dama*) and red deer (*Cervus elaphus*) from Germany [1,34,70].

In *I. ricinus,* collected from the pasture from flock B, the *16S rRNA* gene variants of *A. phagocytophilum* 16S-21 (X) and 16S-22-(Y) were characterized, but both variants were not identified from the ovine samples of flock B. Instead, variant ‘X’ was found in one sheep sample from flock E. Both variants are considered to be apathogenic [70]. Moreover, variants ‘X’ and ‘Y’ were regularly identified in samples from roe deer (*Capreolus capreolus*) and ticks, and to a lesser extent in mouflon (*Ovis musimon*), sika deer (*Cervus nippon*), and fallow deer (*Dama dama*) [1,39,70].

The *16S rRNA* analysis has been previously discussed as less suitable for exploring the molecular epidemiology of *A. phagocytophilum* due to its low discriminatory power. The variant analysis based on the *16S rRNA* gene identified only three-point mutations among the obtained sequences from sheep and ticks. These results confirm the low genetic evolution of this marker being considered not informative enough to undertake heterogeneity analyses for *A. phagocytophilum* in Europe [34,75,76].

Based on the *groEL* gene, six different variants have been identified in lambs and ticks, additionally yet unknown variants being found in one lamb from each of the flocks A and B, and two ticks from a pasture from flock B. The *groEL* analysis showed a higher heterogeneity between the examined samples compared to the *16S rRNA* analysis confirming the intermediate genetic variability of *groEL* gene [34,76]. Variant g-2(B) found in lambs seems to have a broad tropism in wild and domestic animals, but also a zoonotic potential being detected in a patient with HGA from Slovenia [77]. Variant g-24 was also found in an HGA case in Belgium [76], while the BLAST analysis indicated that g-24 isolates of our study match to sequences detected in racoons (*Procyon lotor*; GenBank access. no.: MG670108), *I. ricinus* ticks (GenBank access. no.: KF312360), sheep (GenBank access. no.: EU860089) or red deer (*Cervus elaphus*; GenBank access. no.: HM057225), suggesting a large range of host species that can act as reservoir hosts for this variant, but not all infected animal/tick species are suitable as reservoir. The variants g-4(D) and g-7(G) described in ticks from this study have been previously identified in roe deer (*Capreolus capreolus*) samples [78,79,80] and it has been suggested that these variants circulate between ticks and roe deer (*Capreolus capreolus*). The *groEL* point mutations (40 in total) observed in this study between the different isolates from sheep and ticks did not cause amino acid changes, which means a lack of structure modification to the resulting protein. This might suggest that the pathogenicity does not differ between *A. phagocytophilum groEL* variants.

The variant analysis based on *msp4* gene detected four known variants: m4-5 (I), m4-18 and m4-20 in lambs and m4-13 in ticks. Six sheep samples and one tick sample showed unknown variants. With a total of 28-point mutations between isolates, two of these mutations differentiate variants between tick and sheep samples, one mutation at position 79 causes amino acid change, from isoleucine (hydrophobic) in ticks to valine (hydrophobic) in sheep. The major surface proteins also encoded by *msp4* gene are in constant interaction with the host and vector immune system which can result, due to the selective pressure, in the fast evolution of the *A. phagocytophilum* strains. This fast evolution facilitates the existence of a broad range of circulating *msp4* variants and the detection of yet unknown variants, as was the case in the current study [81].

Selenium deficiency and/or endoparasitism were diagnosed in all flocks and might concur to the ill thriftiness [82,83]. Both diseases are regularly diagnosed in sheep flocks [83,84,85] and have an impact on hematological and biochemical values [42,43,44]. Reticulocytes were not determined, and thrombocytopenia was not confirmed by examination of blood smears due to operational reasons. The primary aim of this field study was to investigate the cause of ill thriftiness, and therefore no clinical healthy animals were included as control groups. Moreover, a small sample size of sheep in each flock was examined and this hampered the calculation of a reliable intra-flock prevalence of *Anaplasma* spp. infection. These limitations narrow the significance of our study, but our investigations provide important insights in the dissemination and genetic diversity of *A. phagocytophilum* and *A. ovis* in sheep from Germany.

## 4. Materials and Methods

### 4.1. Animals’ History, Clinical Examination and Sampling

The five sheep flocks (A-E) consisted of approximately 800 to 1000 Merino ewes in each flock. They were located in the district of Lower Franconia in the German federal state of Bavaria and the extensive pastures were situated in the upland areas of Spessart and southern Rhön. The altitude ranged from 230 to 600 m above sea level. In May 2020, *A. phagocytophilum* was already identified in flocks A and B, in samples from lambs with high fever and from ewes in poor condition during routine diagnostics. In July 2020, all five shepherds reported ill thrift in lambs aged between three to six months. In addition, several ewes were unthrifty in flock A. The term ‘ill thriftiness’ is defined as poor growth rate of lambs and gimmers and loss of body condition in adult sheep [85]. The flocks were then visited in July 2020 to investigate the cause of illness. Twelve ill thrift lambs and three ewes in poor condition from flock A, and 10 unthrifty lambs from each of the other flocks (B-E) were clinically examined. The clinical inspection included body temperature, body condition score [BCS; five-unit scale, (1 = spinal and transverse processes are sharp and no fat is detectable on the loin area to 5 = obese)] [86], color of the conjunctiva [FAMACHA©Score, five-unit scale, (1 = red, non-anemic) to 5 = white, severely anemic)] [86], nasal discharge, lacrimal secretion and observed wheezing/coughing during the examination. The results of the clinical inspection were recorded. EDTA-blood and blood serum samples (Vacuette^®^, Greiner Bio-One, Frickenhausen, Germany) were taken from the *Vena jugularis*. Fecal samples were collected directly from the rectum. Blood and fecal samples were cooled and sent to the Clinic for Swine and Small Ruminants for further processing the next day. In November 2020, the shepherd from flock A reported that one of his breeding rams was suffering from anorexia, dullness, fever and had a slight pale appearance of his conjunctiva (FAMACHA©Score 4). An EDTA blood sample was taken by the local veterinary practitioner and sent to the Clinic for Swine and Small Ruminants for *Anaplasma* diagnostics.

### 4.2. Clinicopathological and Fecal Parasitological Examinations

#### 4.2.1. Complete Blood Cell Count and Microscopical Examinations of Blood Smears

Complete blood count was performed with EDTA-blood and the automated analyzer Celltac Alpha VET MEK-6550 (Nihon Kohden, Tokyo, Japan). Differential blood cell counts were analyzed by microscopical evaluation of blood smears prepared with a modified May-Grünwald Giemsa staining [87]. Briefly, each blood smear was fixed by May-Grünwald solution (May-Grünwald’s eosine-methylene blue solution modified, Merck, Darmstadt, Germany) for 5 min in a cuvette followed by washing with buffered distilled water (pH = 7.2) in a second cuvette and stained with 6% buffered Giemsa solution (Giemsa’s azur eosin methylene blue solution, Merck, Darmstadt, Germany) for 20 min in a third cuvette. After washing again with buffered distilled water (pH = 7.2) and drying, the slides were observed using a light microscopy at 630-x magnification. In total, 200 leukocytes were differentiated in each blood smear according to their morphological features. The outcomes were converted to Giga per litre (G/L; [Total amount of leucocytes x counted amount of each leukocyte type / 100]).

The results were evaluated based on reference values of Lepherd and colleagues [88] for lambs and Ganter [89] for ewes. The diagnosis of anemia (see Table S1) and the grade of anemia severity was based on hemoglobin concentration. Moreover, the severity of anemia in lambs was characterized according to a modified WHO classification [90] as follows: ≥ 105 g/L: non-anemic; 90–104 g/L: mild; 75–89 g/L: moderate; 60–74 g/L: severe; < 60 g/L: life-threatening. Anemia was also classified based on values of mean corpuscular volume (MCV) and mean corpuscular hemoglobin concentration (MCHC).

#### 4.2.2. Biochemical Profile

Total protein, albumin, bilirubin, creatinine, aspartate aminotransferase (AST), creatine kinase (CK) and glutamate dehydrogenase (GDH) concentrations were determined with the chemistry Cobas Mira Plus (Hoffmann La Roche, Basel, Switzerland). Creatine kinase (CK) was analyzed only for animals with elevated AST values. Globulin levels were calculated by subtraction of albumins from total proteins. Serum selenium levels were analyzed by graphite furnace atomic absorption spectroscopy (GFAAS, SOLAAR M, ThermoFisher Scientific, Dreieich, Germany) as previously described [91]. The biochemical outcomes were assessed according to the literature [88,89,92].

#### 4.2.3. Fecal Parasitological Examination

A modified McMaster method was performed to determine the egg per gram of feces (EpG) of gastrointestinal nematodes and the oocyst per gram of feces (OpG) of *Eimeria* spp., respectively. In brief, four grams of feces were weighed and mixed with 60 mL of saturated salt solution (specific gravity 1.2). The mixture was passed through a wire mesh screen and the retentate discarded. After mixing, the sample of the filtrate was examined in a two-chambered McMaster slide (E. Krecek, Onderstepoort, South Africa) where each egg counted represented 50 eggs/oocysts per gram. *Eimeria* spp. were characterized from pooled fecal samples from the examined lambs in each flock as previously described by Eckert et al. [93]. The percentage of *E. ovinoidalis,* determined from a pool sample was reported as the most pathogenic *Eimeria* species for lambs [82].

### 4.3. Ticks Collection, Identification and Storage

Questing ticks were collected in July 2020 by dragging and flagging a 1 m^2^ white cotton material on three pastures previously grazed by sheep from flock B. After collection, ticks were subjected to morphological identification using taxonomical identification keys [94] then stored at −80 °C until further processing. In November 2020, the shepherd from flock A sent in seven ticks collected from three ewes. The ewes came from the same flock as the sick ram.

### 4.4. Anaplasma phagocytophilum and Anaplasma ovis Laboratory Investigations

#### 4.4.1. Antibody Detection

Antibodies against *Anaplasma* spp. were determined by two serological tests. Serum samples from nine ill thrift lambs and two unthrift ewes (Flock A and B) were sent in May 2020 and included in the serological examination.

*Anaplasma* spp. antibodies were detected in sheep’s serum by a competitive ELISA (*Anaplasma* Antibody Test Kit, cELISA v2, VMRD, Pullman, USA). The cELISA was applied according to the manufacturer’s instructions and samples having ≥30% inhibition was considered positive. The cELISA uses recombinant MSP (rMSP5) to detect antibodies to *A. marginale*, *A. ovis* and *A. centrale* in cattle. Ovine sera were already analyzed with this assay to determine *Anaplasma* spp. antibodies in areas where *A. ovis* and *A. phagocytophilum* are present [28,60].

*Anaplasma phagocytophilum* antibodies were determined by a semi-quantitative IFAT according to the manufacturer’s instructions. Briefly, fluorescein-labeled anti-sheep IgG (MegaFLUO^®^ VET, Megacor Diagnostik GmbH, Hoerbranz, Austria) was used to detect IgG antibody-antigen complexes. All serum and controls were tested on microscope slides (MegaFLUO^®^ *ANAPLASMA phagocytophilum*, Megacor Diagnostik GmbH, Hoerbranz, Austria), coated with *A. phagocytophilum* antigens. Serum samples were diluted 1:40, 1:80, 1:160, 1:320 and 1:640 with PBS (pH 7.2). A volume of 20 µL of each sample dilution was applied to the slide and incubated for 30 min at 37 °C. Unbound antibodies were removed by washing with PBS (pH 7.2) and Aqua bidest. After drying, 20 µL of the species-specific fluorescein-conjugated antibodies were applied, and the slide was incubated for further 30 min at 37 °C under light protection. Unbound antibodies were again removed by washing with PBS (pH 7.2) and Aqua bidest. Next, a mounting medium (Eukitt^®^ Quick-hardening mounting medium, Sigma-Aldrich, Steinheim, Germany) was put on the slide, followed by a coverslip. Finally, the slides were observed using UV light microscopy at 400-fold magnification. Seropositive samples were identified by the presence of fluorescence from ≥1:40 [95].

#### 4.4.2. Microscopical Detection in Blood Smears

Stained blood smears (see 4.2.1) were observed for morulae and inclusion bodies using a light microscopy at 630-magnification. In total, 30 visual fields were examined per slide. Animals tested positive for *A. phagocytophilum* if morulae in leucocytes were present. Additionally, the detection of inclusion bodies in erythrocytes was considered as *A. ovis* positive.

#### 4.4.3. DNA Detection in Blood and Ticks

Besides the 55 EDTA-blood samples collected in July 2020 from flock A-E, specimens were sent for analysis from flock A and B in May and November 2020. These 12 samples were also included in the molecular examination.

##### DNA Extraction

Blood samples collected on EDTA-containing vacutainer tubes were further processed using 200 µL blood aliquots from each sample to extract total DNA with QIAamp DNA Mini kit (Qiagen, Hilden, Germany) and NucleoMag^®^ VET kit (Macherey-Nagel, Düren, Germany) with the King Fisher^®^ Flex Purification system (ThermoFisher, Darmstadt, Germany), following the manufacturer’s instructions. Total DNA was eluted in 100 μL of elution buffer and stored at −20 °C until further use.

Prior to the extraction of DNA, tick samples were processed for tissue homogenization. Adult ticks from the pasture were processed individually, while nymphs from the pasture were pooled in six to 10 ticks per pool, pooling being conducted based on the collection site. The seven engorged ticks collected from the sheep were also pooled according to their species. For tissue lysing the samples, ticks were added to 2 mL tubes, each tube containing two sterile 4 mm metal beads and 300 µL sterile phosphate buffered saline. Samples were lysed using Tissue Lyser II (Qiagen, Hilden, Germany) twice for 1 min, at 30 Hz. Then, after centrifugation of samples at 211 g for 1 min, 200 µL of tick homogenate were transferred to 1.5 mL tubes and stored at −20 °C for DNA extraction. The isolation of DNA was conducted using NucleoMag^®^ VET kit (Macherey-Nagel, Düren, Germany) and the King Fisher^®^ Flex Purification system (ThermoFisher, Darmstadt, Germany), following the manufacturer’s instructions. Elution of DNA was conducted in 100 μL of elution buffer then samples were stored at −20 °C until further use.

##### Real Time PCR

DNA extracted from sheep blood and ticks was included in qPCR reactions for the amplification of *A. phagocytophilum* and *A. ovis.* Each PCR assay included specific primers and probe targeting a 77 bp fragment of *msp2* gene for *A. phagocytophilum* and a 92 bp fragment of *msp4* gene for *A. ovis*, respectively (Table 4) [96,97]. The fluorogenic probes were synthetized with a 6-carboxy-fluorescein (FAM) reporter molecule attached to the 5′ end and a Black Hole Quencher 1 (BHQ1) at the 3′ end. The amplification was performed in a total volume of 25 µL reaction mix using ITaq Universal Probes Supermix (BioRad Laboratory Inc., Munich, Germany) and CFX-96 Real-Time system (BioRad Laboratory Inc., Munich, Germany). The reaction mix included 10 µL of DNA, 12.5 µL of iTaq^TM^ Supermix (2×), 900 nM of each forward and reverse primers, 120 nM of probe and 1.75 µL of nuclease-free water in a final volume of 25 µL. The thermal profile had an initial denaturation at 95 °C for 5 min followed by 40 cycles of denaturation at 95 °C for 5 s and annealing/elongation at 60 °C for 30 s. Each reaction included positive (DNA of *A. phagocytophilum* from ticks, DNA of *A. ovis* from sheep) and negative (molecular grade water) controls. No internal control was used to assess the presence of PCR inhibitors.

##### Nested PCR of Real Time *Anaplasma ovis* Postive Blood Samples

Positive samples for *A. ovis* in the qPCR reaction were also amplified by nested PCR using specific primers for *msp4* target gene (Table 4) [98]. Confirmation of positive amplicons was obtained as described for the *A. phagocytophilum* PCR products.

##### Sequence Analysis

*Anaplasma phagocytophilum* isolates from all sheep and five out of nine ticks amplified in the qPCR reaction were further analyzed for the identification of the circulating genotypes. For this purpose, three different targets were amplified (*16S rRNA*, *msp4* and *groEL* genes) using specific primer pairs (Table 4). The PCR reactions were performed using GoTaq^®^ G2 Flexi DNA Polymerase kit (Promega, Walldorf, Germany) and C1000 Thermal Cycler (BioRad Laboratory Inc., Feldkirchen, Germany) [34]. Confirmation of the positive PCR products was made by separating the amplicons on a 1.5% agarose gel stained with Roti^®^-GelStain Red (Carl Roth GmbH, Karlsruhe, Germany) followed by gel visualization with ChemiDoc™ MMP Imaging system (Bio-Rad Laboratories, Feldkirchen, Germany).

Positive PCR products were purified with NucleoSEQ^®^ kit (Macherey Nagel, Düren, Germany) following the manufacturer’s instructions. After purification, samples were included in a sequencing PCR, in 10 µL reaction mix: 1 µL of 5× Sequence buffer, 2 µL Big Dye Ready Reaction Mix (Thermo Fischer, Darmstadt, Germany), 1 µL of forward or reverse primer (10 µM), 5 µL of molecular grade water and 1 µL of the purified amplicon. The thermal conditions were as follows: denaturation at 96 °C for 1 min then 25 cycles of denaturation at 96 °C for 10 s, annealing at the specific annealing temperature for each primer for 5 s (Table 4), elongation at 60 °C with duration varying with the length of the products. The obtained products were then purified with NucleoSEQ kit (Macherey-Nagel, Düren, Germany) and 15 µL of each purified product were mixed with 15 µL of highly deionized (Hi-Di) formamide in a 1.5 mL tube and sequenced on an ABI PRISM^®^ 3130 sequencer.

Following sequencing, the obtained sequences were viewed and edited using Geneious 11.1.5 (Biomatters, Auckland, New Zealand) and then compared with sequences available in the GenBank database using BLASTn (http://www.ncbi.nlm.nih.gov.library.vu.edu.au/BLAST/ (accessed on 25 June 2021)).

### 4.5. Statistical Analysis

Fisher’s exact tests were used to investigate the influence of *Anaplasma* spp. infection on clinical signs, hematological and biochemical abnormalities from 55 sheep sampled in July 2020. Sheep were considered *Anaplasma* spp. positive if antibodies and/or DNA were detected. Results *p* < 0.05 were assessed as significant. Descriptive statistics of outcomes from fecal parasitological examinations were performed and data are expressed as mean ± standard error (GraphPad Prism 9, Cypress, CA, USA).

## 5. Conclusions

*Anaplasma phagocytophilum* seems to be widely disseminated in sheep flocks from Lower Franconia and different genetic variants circulate sympatrically in this geographic region, whereas *A. ovis* occurred only in a restricted area. However, the focal distribution of *Dermacentor* spp. across Germany and the increase in very hot dry summer months will rise the clinical cases of *A. ovis* [22]. Our findings underline the fact, that both tick-borne pathogens are emerging in German sheep flocks. Further research is essential to investigate the dissemination, vectors and clinical impact of both *Anaplasma* species, particularly in case of comorbidities. This is also in line with the One Health concept as *A. phagocytophilum* and *A. ovis* can have adverse effects on human health, and the epidemiology of HGA is still poorly understood in Europe [6].

## Figures and Tables

**Table 1:** Detection of anti-*Anaplasma* spp. (ELISA cut off: inhibition ≥30%), and anti-*Anaplasma phagocytophilum* (IFAT cut off dilution: 1:40) antibodies, and quantification cycle value of DNA target fragments of *A. phagocytophilum* and *A. ovis* (qPCR) in sheep from the five flocks sampled between May and November 2020.

Flock	Collection Date	Sample ID	cELISA	IFAT	*A. phagocytophilum* qPCR	*A. ovis* qPCR
**A**	May 2020	T668.1 ewe	**81.7**	**≥1:640**	**23.5**	**33.3**
		T668.2 lamb	**46.9**	**1:40**	**17.9**	**-**
		T668.3 lamb	**52.1**	**1:320**	**21.5**	**-**
	July 2020	T817.1 lamb	**92.4**	**≥1:640**	**-**	**23.3**
		T817.2 lamb	23.1	**≥1:640**	**33.9**	-
		T817.3 lamb	**64.8**	**≥1:640**	**33.1**	-
		T817.4 lamb	25.2	**1:40**	**-**	**-**
		T817.5 lamb	**84**	**1:320**	**-**	**29.3**
		T817.6 lamb	**51.6**	**1:80**	**-**	-
		T817.7 lamb	**86.6**	**1:160**	**25.4**	-
		T817.8 lamb	22.1	**≥1:640**	-	-
		T817.9 lamb	**44.8**	**1:320**	-	-
		T817.10 lamb	**71.3**	**≥1:640**	26.0	-
		T817.11 ewe	**94.1**	**≥1:640**	-	**20.6**
		T817.12 ewe	**87.6**	**≥1:640**	-	**-**
		T817.13 ewe	**92.6**	**1:160**	-	**21**
		T817.14 lamb	**42**	**1:40**	-	-
		T817.15 lamb	**31.5**	**1:160**	-	-
	November 2020	T1339 ram	n/a	n/a	-	**18**
**B**	May 2020	T642.1 lamb	−8.9	1:320	36.2	-
		T642.2 lamb	−2.9	-	-	-
		T642.3 lamb	−11.1	1:140	23.5	-
		T642.4 lamb	−14.5	-	-	-
		T642.5 lamb	−48.2	-	-	
		T642.8 ewe	**54.6**	**1:160**	**-**	-
		T642.9 lamb	−5.1	**-**	**-**	-
		T642.10 lamb	−4.4	**-**	**-**	-
	July 2020	T820.1 lamb	**59.9**	**≥1:640**	**37.0**	-
		T820.2 lamb	−0.7	-	-	-
		T820.3 lamb	**48.5**	**≥1:640**	-	-
		T820.4 lamb	12.9	-	-	-
		T820.5 lamb	**81**	**≥1:640**	**29.1**	-
		T820.6 lamb	**55.9**	**≥1:640**	**-**	-
		T820.7 lamb	**60.2**	**≥1:640**	**20.8**	-
		T820.8 lamb	**77.1**	**≥1:640**	21.7 *	-
		T820.9 lamb	**63.9**	**≥1:640**	**-**	-
		T820.10 lamb	**44.9**	**1:320**	**32.7**	-
**C**	July 2020	T819.1 lamb	**38.6**	**≥1:640**	**-**	-
		T819.2 lamb	26.7	-	-	-
		T819.3 lamb	22.9	-	-	-
		T819.4 lamb	12.3	-	-	-
		T819.5 lamb	**59.1**	-	-	-
		T819.6 lamb	**38.1**	-	-	-
		T819.7 lamb	19.2	-	-	-
		T819.8 lamb	21	-	-	-
		T819.9 lamb	7.2	-	-	-
		T819.10 lamb	29.5	**≥1:640**	16.6 *	-
**D**	July 2020	T818.1 lamb	21.2	-	-	-
		T818.2 lamb	**36.3**	**1:40**	-	-
		T818.3 lamb	**38.8**	**≥1:640**	-	-
		T818.4 lamb	27.5	**≥1:640**	-	-
		T818.5 lamb	23	**≥1:640**	-	-
		T818.6 lamb	12.3	-	-	-
		T818.7 lamb	12.7	-	-	-
		T818.8 lamb	18.6	-	-	-
		T818.9 lamb	**49.7**	**≥1:640**	**28.1**	-
		T818.10 lamb	15.5	-	-	-
**E**	July 2020	T921.1 lamb	9.2	-	-	-
		T921.2 lamb	−4.5	-	**35.5**	-
		T921.3 lamb	−33.2	-	-	-
		T921.4 lamb	−9.5	-	-	-
		T921.5 lamb	19.1	-	-	-
		T921.6 lamb	13.8	-	-	-
		T921.7 lamb	14.7	-	-	-
		T921.8 lamb	**31.9**	-	-	-
		T921.9 lamb	12.4	-	-	-
		T921.10 lamb	−11.1	-	-	-

Positive results are indicated in bold. * *A. phagocytophilum* morulae were identified in blood smears; n/a = not applicable.

**Table 2:** Analyzed sequences of *Anaplasma phagocytophilum* in sheep from five flocks with ill thriftiness. Cq = cycle quantification; n/a = not applicable.

Flock	Sample ID	Sample Date	Cq-Value	*16S rRNA*	*groEL*	*msp4*
**A**	T668.1 ewe	May 2020	23.5	16S-16 (S)	g-35	New (shorter than other sequences (25 nt at beginning)
	T668.2 lamb		17.9	16S-16 (S)	g-35	new
	T668.3 lamb		21.5	16S-16 (S)	g-35	new
	T817.2 lamb	July 2020	33.9	16S-2 (B)	g-2 (B)	m4-20
	T817.3 lamb		33.1	n/a	n/a	n/a
	T817.7 lamb		25.4	16S-16 (S)	g-13	m4-18
	T817.10 lamb		26.0	16S-20 (W)	new	new
**B**	T642.1 lamb	May 2020	36.2	n/a	n/a	n/a
	T642.3 lamb		23.5	16S-16 (S)	new	new
	T820.1 lamb	July 2020	37.0	16S-20 (W)	Not clear, ambiguous pos. 473 (W)	m4-5 (I)
	T820.5 lamb		29.1	16S-16 (S)	g-24	new
	T820.7 lamb		20.8	16S-16 (S)	g-35	New (shorter than other sequences (25 nt at beginning)
	T820.8 lamb		21.7	16S-20 (W)	new	m4-5 (I)
	T820.10 lamb		32.7	16S-20 (W)	New, ambiguous pos. 515 (K)	n/a
**C**	T819.10 lamb	July 2020	16.6	16S-20 (W)	g-24	m4-5 (I)
**D**	T818.9 lamb	July 2020	28.1	16S-2 (B)	g-2 (B)	m4-20
**E**	T921.2 lamb	July 2020	35.5	16S-21 (X)	n/a	new

**Table 3:** Analyzed sequences of *Anaplasma phagocytophilum* in five pools of questing *Ixodes ricinus* nymphs from two pastures of flock B.

Flock	Sample ID	Sample Date	Cq-value	16S rRNA	groEL	msp4
**B**	ET-9	July 2020	28	16S-21 (X)	new	m4-13 (N)
	ET-10		26.6	16S-21 (X)	g-7 (G)	m4-13 (N)
	ET-13		27.3	16S-21 (X)	new	m4-13 (N)
	HT-22		23.2	16S-22 (Y)	g-4 (D)	m4-13 (N)
	HT-24		29	16S-22 (Y)	n/a	new

**Table 4:** Primers used for amplification of *Anaplasma phagocytophilum* and *Anaplasma ovis*.

Target Gene	Reaction	Sequence (5′-3′)	Amplicon Size (bp)	Annealing	**Reference**
***Anaplasma phagocytophilum***					
***msp2***	qPCR	ApMSP2f: TGGAAGGTAGTGTTGGTTATGGTATT ApMSP2r: TTGGTCTTGAAGCGCTCGTA ApMSP2p: TGGTGCCAGGGTTGAGCTTGAGATTG	77	60 °C	[96]
***16S rRNA***	Nested PCR	First PCR:			[99]
		Ge3a: CACATGCAAGTCGAACGGATTATTC	932	55 °C	
		Ge10r: TTCCGTTAAGAAGGATCTAATCTCC			
		Nested PCR *:			
		Ge9f: AACGGATTATTCTTTATAGCTTGCT	546	55 °C	
		Ge2: GGCAGTATTAAAAGCAGCTCCAGG			
***msp4***	Nested PCR	First PCR:			[100]
		Msp4AP5: ATGAATTACAGAGAATTGCTTGTAGG	849	54 °C	
		Msp4AP3: TTAATTGAAAGCAAATCTTGCTCC			
		TATG			
		Nested PCR *:			
		Msp4f: CTATTGGYGGNGCYAGAGT			
		Msp4r: GTTCATCGAAAATTCCGTGGTA	362	54 °C	
***groEL***	Nested PCR	First PCR:			[101]
		EphplgroEL-F: ATGGTATGCAGTTTGATCGC	624	55 °C	
		EphplgroEL-R: TCTACTCTGTCTTTGCGTTC			
		Nested PCR *:			
		EphplgroEL-F: ATGGTATGCAGTTTGATCGC	573	55 °C	
		EphgroEL-R: TTGAGTACAGCAACACCACCGGAA			
***Anaplasma ovis***					
***msp4***	qPCR	A_ov_msp4_F: TCATTCGACATGCGTGAGTCAA_ov_msp4_R: TTTGCTGGCGCACTCACATCA_ov_msp4_P: AGCAGAGAGACCTCGTATGTTAGAGGC	92	60 °C	[97]
***msp4***	Nested PCR	First PCR:			[98]
		M-OM F: GGGAGCTCCTATGAATTACAGAGAATTGTTTAC	870	60 °C	
		M-OM R: CCGGATCCTTAGCTGAACAGGAATCTTGC			
		Nested PCR *:			
		M-OV F: TGAAGGGAGCGGGGTCATGGG	346	60 °C	
		M-OV R: GGTAATTGCAGCCAGGGACTCT			

* Primer pairs used in sequencing PCR.

## Data Availability

The data that support the findings of this study are available from the corresponding author upon request.

## Supplementary Materials

The following are available online at https://www.mdpi.com/article/10.3390/pathogens10101298/s1, Table S1: Raw data from the serological (cELISA and IFAT) and PCR [*Anaplasma phagocytophilum* (Ap), *Anaplasma ovis* (Ao)] tests, clinical and clinicopathological (CBC and biochemical profile) examination, fecal parasitological evaluation of 55 ill thrift sheep sampled in July 2020. Reference values of parameters included in the clinicopathological examination are reported at the top of each column in italics for sheep and bold for lambs. Table S2. Raw data of clinical, hematological and biochemical findings from *Anaplasma* spp. positive (cELISA and/or PCR, *n* = 34) and *Anaplasma* spp. negative (*n* = 21) sheep from five sheep flocks sampled in July 2020. **p* < 0.05 is considered as significant. Table S3: BLAST results retrieved for *Anaplasma phagocytophilum 16S rRNA*, *groEl* and *msp4* sequences. Figure S1: Phylogenetic tree of *Anaplasma phagocytophilum 16S rRNA* sequences derived from sheep and *Ixodes ricinus* ticks. Figure S2: Phylogenetic tree of *Anaplasma phagocytophilum groEL* sequences derived from sheep and *Ixodes ricinus.* Figure S3: Phylogenetic tree of *Anaplasma phagocytophilum msp4* sequences from sheep and *Ixodes ricinus*.

## References

[1] Kauffmann M., Rehbein S., Hamel D., Lutz W., Heddergott M., Pfister K., Silaghi C. (2017). *Anaplasma phagocytophilum* and *Babesia* spp. in roe deer (*Capreolus capreolus*), fallow deer (*Dama dama*) and mouflon (*Ovis musimon*) in Germany. Mol. Cell. Probes.

[2] de la Fuente J., Ruiz-Fons F., Naranjo V., Torina A., Rodríguez O., Gortázar C. (2008). Evidence of *Anaplasma* infections in European roe deer (*Capreolus capreolus*) from southern Spain. Res. Vet. Sci..

[3] Scharf W., Schauer S., Freyburger F., Petrovec M., Schaarschmidt-Kiener D., Liebisch G., Runge M., Ganter M., Kehl A., Dumler J.S. (2011). Distinct host species correlate with *Anaplasma phagocytophilum* ankA gene clusters. J. Clin. Microbiol..

[4] Víchová B., Majláthová V., Nováková M., Stanko M., Hviščová I., Pangrácová L., Chrudimský T., Čurlík J., Peťko B. (2014). *Anaplasma* infections in ticks and reservoir host from Slovakia. Infect. Genet. Evol..

[5] Stuen S. (2016). Haemoparasites in small ruminants in European countries: Challenges and clinical relevance. Small Rumin. Res..

[6] Matei I.A., Estrada-Peña A., Cutler S.J., Vayssier-Taussat M., Varela-Castro L., Potkonjak A., Zeller H., Mihalca A.D. (2019). A review on the eco-epidemiology and clinical management of human granulocytic anaplasmosis and its agent in Europe. Parasites Vectors.

[7] Stuen S., Granquist E., Silaghi C. (2013). *Anaplasma phagocytophilum*—A widespread multi-host pathogen with highly adaptive strategies. Front. Cell. Infect. Microbiol..

[8] Choi K.S., Garyu J., Park J., Dumler J.S. (2003). Diminished adhesion of *Anaplasma phagocytophilum*-infected neutrophils to endothelial cells is associated with reduced expression of leukocyte surface selectin. Infect. Immun..

[9] Stuen S., Van De Pol I., Bergström K., Schouls L.M. (2002). Identification of *Anaplasma phagocytophila* (formerly *Ehrlichia phagocytophila*) variants in blood from sheep in Norway. J. Clin. Microbiol..

[10] Almazán C., Fourniol L., Rouxel C., Alberdi P., Gandoin C., Lagrée A.-C., Boulouis H.-J., de la Fuente J., Bonnet S.I. (2020). Experimental *Ixodes ricinus*-sheep cycle of *Anaplasma phagocytophilum* NV2Os propagated in tick cell cultures. Front. Vet. Sci..

[11] Grøva L., Olesen I., Steinshamn H., Stuen S. (2011). Prevalence of *Anaplasma phagocytophilum* infection and effect on lamb growth. Acta Vet. Scand..

[12] Stuen S., Bergström K., Palmér E. (2002). Reduced weight gain due to subclinical *Anaplasma phagocytophilum* (Formerly *Ehrlichia phagocytophila*) infection. Exp. Appl. Acarol..

[13] Stuen S., Scharf W., Schauer S., Freyburger F., Bergström K., von Loewenich F.D. (2010). Experimental infection in lambs with a red deer (*Cervus elaphus*) isolate of *Anaplasma phagocytophilum*. J. Wildl. Dis..

[14] Gokce H.I., Woldehiwet Z. (1999). Differential haematological effects of tick-borne fever in sheep and goats. Zentralbl. Veterinarmed. B.

[15] Daniel R.G., Carson A., Evans C., Cookson R., Wessels M. (2016). Pathological observations of tick-borne fever and intercurrent bacterial infections in lambs. Vet. Rec. Case Rep..

[16] Overås J., Lund A., Ulvund M.J., Waldeland H. (1993). Tick-borne fever as a possible predisposing factor in septicaemic pasteurellosis in lambs. Vet. Rec..

[17] Sargison N., Edwards G. (2009). Tick infestations in sheep in the UK. Practice.

[18] Jiménez C., Benito A., Arnal J.L., Ortín A., Gómez M., López A., Villanueva-Saz S., Lacasta D. (2019). *Anaplasma ovis* in sheep: Experimental infection, vertical transmission and colostral immunity. Small Rumin. Res..

[19] Pereira A., Parreira R., Nunes M., Casadinho A., Vieira M.L., Campino L., Maia C. (2016). Molecular detection of tick-borne bacteria and protozoa in cervids and wild boars from Portugal. Parasites Vectors.

[20] Hornok S., Elek V., de la Fuente J., Naranjo V., Farkas R., Majoros G., Földvári G. (2007). First serological and molecular evidence on the endemicity of *Anaplasma ovis* and *A. marginale* in Hungary. Vet. Microbiol..

[21] Chochlakis D., Ioannou I., Tselentis Y., Psaroulaki A. (2010). Human anaplasmosis and *Anaplasma ovis* variant. Emerg. Infect. Dis..

[22] Renneker S., Abdo J., Salih D.E., Karagenç T., Bilgiç H., Torina A., Oliva A.G., Campos J., Kullmann B., Ahmed J. (2013). Can *Anaplasma ovis* in small ruminants be neglected any longer?. Transbound. Emerg. Dis..

[23] Derdáková M., Štefančíková A., Špitalská E., Tarageľová V., Košťálová T., Hrkľová G., Kybicová K., Schánilec P., Majláthová V., Várady M. (2011). Emergence and genetic variability of *Anaplasma* species in small ruminants and ticks from Central Europe. Vet. Microbiol..

[24] Friedhoff K.T. (1997). Tick-borne diseases of sheep and goats caused by *Babesia*, *Theileria* or *Anaplasma* spp.. Parassitologia.

[25] Hornok S., de la Fuente J., Biró N., Fernández de Mera I.G., Meli M.L., Elek V., Gönczi E., Meili T., Tánczos B., Farkas R. (2011). First molecular evidence of *Anaplasma ovis* and *Rickettsia* spp. in keds (Diptera: Hippoboscidae) of sheep and wild ruminants. Vector Borne Zoonotic Dis..

[26] Zhang Q.X., Wang Y., Li Y., Han S.Y., Wang B., Yuan G.H., Zhang P.Y., Yang Z.W., Wang S.L., Chen J.Y. (2021). Vector-borne pathogens with veterinary and public health significance in *Melophagus ovinus* (Sheep Ked) from the Qinghai-Tibet Plateau. Pathogens.

[27] Lacasta D., Ferrer L.M., Sanz S., Labanda R., González J.M., Benito A., Ruiz H., Rodríguez-Largo A., Ramos J.J. (2020). Anaplasmosis outbreak in lambs: First report causing carcass condemnation. Animals.

[28] Torina A., Galindo R.C., Vicente J., Di Marco V., Russo M., Aronica V., Fiasconaro M., Scimeca S., Alongi A., Caracappa S. (2010). Characterization of *Anaplasma phagocytophilum* and *A. ovis* infection in a naturally infected sheep flock with poor health condition. Trop. Anim. Health Prod..

[29] Yasini S.P., Khaki Z., Rahbari S., Kazemi B., Salar Amoli J., Gharabaghi A., Jalali S.M. (2012). Hematologic and clinical aspects of experimental ovine anaplasmosis caused by *Anaplasma ovis* in Iran. Iran. J. Parasitol..

[30] Battilani M., De Arcangeli S., Balboni A., Dondi F. (2017). Genetic diversity and molecular epidemiology of *Anaplasma*. Infect. Genet. Evol..

[31] Jaarsma R.I., Sprong H., Takumi K., Kazimirova M., Silaghi C., Mysterud A., Rudolf I., Beck R., Földvári G., Tomassone L. (2019). *Anaplasma phagocytophilum* evolves in geographical and biotic niches of vertebrates and ticks. Parasites Vectors.

[32] Lagrée A.C., Rouxel C., Kevin M., Dugat T., Girault G., Durand B., Pfeffer M., Silaghi C., Nieder M., Boulouis H.J. (2018). Co-circulation of different *A. phagocytophilum* variants within cattle herds and possible reservoir role for cattle. Parasites Vectors.

[33] Langenwalder D.B., Silaghi C., Nieder M., Pfeffer M., von Loewenich F.D. (2020). Co-infection, reinfection and superinfection with *Anaplasma phagocytophilum* strains in a cattle herd based on ankA gene and multilocus sequence typing. Parasites Vectors.

[34] Silaghi C., Liebisch G., Pfister K. (2011). Genetic variants of *Anaplasma phagocytophilum* from 14 equine granulocytic anaplasmosis cases. Parasites Vectors.

[35] Silaghi C., Nieder M., Sauter-Louis C., Knubben-Schweizer G., Pfister K., Pfeffer M. (2018). Epidemiology, genetic variants and clinical course of natural infections with *Anaplasma phagocytophilum* in a dairy cattle herd. Parasites Vectors.

[36] Silaghi C., Hamel D., Thiel C., Pfister K., Passos L.M., Rehbein S. (2011). Genetic variants of *Anaplasma phagocytophilum* in wild caprine and cervid ungulates from the Alps in Tyrol, Austria. Vector Borne Zoonotic Dis..

[37] Ladbury G.A.F., Stuen S., Thomas R., Bown K.J., Woldehiwet Z., Granquist E.G., Bergström K., Birtles R.J. (2008). Dynamic transmission of numerous *Anaplasma phagocytophilum* genotypes among lambs in an infected sheep flock in an area of anaplasmosis endemicity. J. Clin. Microbiol..

[38] Drehmann M., Springer A., Lindau A., Fachet K., Mai S., Thoma D., Schneider C.R., Chitimia-Dobler L., Bröker M., Dobler G. (2020). The spatial distribution of *Dermacentor* ticks (Ixodidae) in Germany—Evidence of a continuing spread of *Dermacentor reticulatus*. Front. Vet. Sci..

[39] Overzier E., Pfister K., Herb I., Mahling M., Böck G., Silaghi C. (2013). Detection of tick-borne pathogens in roe deer (*Capreolus capreolus*), in questing ticks (*Ixodes ricinus*), and in ticks infesting roe deer in southern Germany. Ticks Tick Borne Dis..

[40] Kumar S., Stecher G., Li M., Knyaz C., Tamura K. (2018). MEGA X: Molecular evolutionary genetics analysis across computing platforms. Mol. Biol. Evol..

[41] Tamura K. (1992). Estimation of the number of nucleotide substitutions when there are strong transition-transversion and G+C-content biases. Mol. Biol. Evol..

[42] Huo B., Wu T., Song C., Shen X. (2019). Studies of selenium deficiency in the Wumeng semi-fine wool sheep. Biol. Trace Element Res..

[43] Alam R.T.M., Hassanen E.A.A., El-Mandrawy S.A.M. (2020). Heamonchus contortus infection in sheep and goats: Alterations in haematological, biochemical, immunological, trace element and oxidative stress markers. J. Appl. Anim. Res..

[44] Chapman H. (1974). The effects of natural and artificially acquired infections of coccidia in lambs. Res. Vet. Sci..

[45] Woldehiwet Z. (2010). The natural history of *Anaplasma phagocytophilum*. Vet. Parasitol..

[46] Chirek A., Silaghi C., Pfister K., Kohn B. (2018). Granulocytic anaplasmosis in 63 dogs: Clinical signs, laboratory results, therapy and course of disease. J. Small Anim. Pract..

[47] Franzén P., Aspan A., Egenvall A., Gunnarsson A., Aberg L., Pringle J. (2005). Acute clinical, hematologic, serologic, and polymerase chain reaction findings in horses experimentally infected with a European strain of *Anaplasma phagocytophilum*. J. Vet. Intern. Med..

[48] Foster W.N.M., Cameron A.E. (1968). Thrombocytopenia in sheep associated with experimental tick-borne fever infection. J. Comp. Pathol..

[49] Rankins D.L., Pugh D.G., Pugh D.G., Baird A.N. (2012). Feeding and nutrition. Sheep and Goat Medicine.

[50] Polizopoulou Z. (2010). Haematological tests in sheep health management. Small Rumin. Res..

[51] Katsogiannou E.G., Athanasiou L.V., Christodoulopoulos G., Polizopoulou Z.S. (2018). Diagnostic approach of anemia in ruminants. J. Hell. Vet. Med Soc..

[52] Plummer P.J., Plummer C.L., Still K.M., Pugh D.G., Baird A.N. (2012). Diseases of the respiratory system. Sheep and Goat Medicine.

[53] Siska W.D., Tuttle R.E., Messick J.B., Bisby T.M., Toth B., Kritchevsky J.E. (2013). Clinicopathologic characterization of six cases of equine granulocytic anaplasmosis in a nonendemic area (2008–2011). J. Equine Vet. Sci..

[54] Chow C.K., Chen C.J. (1980). Dietary selenium and age-related susceptibility of rat erythrocytes to oxidative damage. J. Nutr..

[55] Nagababu E., Chrest F.J., Rifkind J.M. (2003). Hydrogen-peroxide-induced heme degradation in red blood cells: The protective roles of catalase and glutathione peroxidase. Biochim. et Biophys. Acta (BBA) Gen. Subj..

[56] Kümper H. (1989). Hypericum poisoning in sheep. Tierarztl. Prax..

[57] Kako M.D., al-Sultan I.I., Saleem A.N. (1993). Studies of sheep experimentally poisoned with *Hypericum perforatum*. Vet. Hum. Toxicol..

[58] Navarre C.B., Baird A., Pugh D., Pugh D.G., Baird A.N. (2012). Diseases of the gastrointestinal system. Sheep and Goat Medicine.

[59] Mason K.L., Gonzalez M.V., Chung C., Mousel M.R., White S.N., Taylor J.B., Scoles G.A. (2017). Validation of an improved *Anaplasma* antibody competitive ELISA for detection of *Anaplasma ovis* antibody in domestic sheep. J. Vet. Diagn. Investig..

[60] Shabana I.I., Alhadlag N.M., Zaraket H. (2018). Diagnostic tools of caprine and ovine anaplasmosis: A direct comparative study. BMC Vet. Res..

[61] Kocan K.M., Busby A.T., Allison R.W., Breshears M.A., Coburn L., Galindo R.C., Ayllón N., Blouin E.F., de la Fuente J. (2012). Sheep experimentally infected with a human isolate of *Anaplasma phagocytophilum* serve as a host for infection of *Ixodes scapularis* ticks. Ticks Tick Borne Dis..

[62] Dreher U.M., de la Fuente J., Hofmann-Lehmann R., Meli M.L., Pusterla N., Kocan K.M., Woldehiwet Z., Braun U., Regula G., Staerk K.D.C. (2005). Serologic cross-reactivity between *Anaplasma marginale* and *Anaplasma phagocytophilum*. Clin. Diagn. Lab. Immunol..

[63] Gorman J.K., Hoar B.R., Nieto N.C., Foley J.E. (2012). Evaluation of *Anaplasma phagocytophilum* infection in experimentally inoculated sheep and determination of *Anaplasma* spp. seroprevalence in 8 free-ranging sheep flocks in California and Oregon. Am. J. Vet. Res..

[64] Stuen S., Bergström K., Petrovec M., Van de Pol I., Schouls L.M. (2003). Differences in clinical manifestations and hematological and serological responses after experimental infection with genetic variants of *Anaplasma phagocytophilum* in sheep. Clin. Diagn. Lab. Immunol..

[65] Thomas R., Birtles R., Radford A., Woldehiwet Z. (2012). Recurrent bacteraemia in sheep infected persistently with *Anaplasma phagocytophilum*. J. Comp. Pathol..

[66] Kiilerich A.M., Christensen H., Thamsborg S.M. (2009). *Anaplasma phagocytophilum* in Danish sheep: Confirmation by DNA sequencing. Acta Vet. Scand..

[67] Stuen S., Pettersen K.S., Granquist E.G., Bergström K., Bown K., Birtles R. (2013). *Anaplasma phagocytophilum* variants in sympatric red deer (*Cervus elaphus*) and sheep in southern Norway. Ticks Tick-borne Dis..

[68] Cabezas-Cruz A., Gallois M., Fontugne M., Allain E., Denoual M., Moutailler S., Devillers E., Zientara S., Memmi M., Chauvin A. (2019). Epidemiology and genetic diversity of *Anaplasma ovis* in goats in Corsica, France. Parasites Vectors.

[69] Lacasta D., Lorenzo M., González J.M., Ruiz de Arcaute M., Benito A., Baselga C., Milian M.E., Lorenzo N., Jiménez C., Villanueva-Saz S. (2021). Epidemiological study related to the first outbreak of ovine anaplasmosis in Spain. Animals.

[70] Silaghi C., Fröhlich J., Reindl H., Hamel D., Rehbein S. (2020). *Anaplasma phagocytophilum* and *Babesia* species of sympatric roe deer (*Capreolus capreolus*), fallow deer (*Dama dama*), sika deer (*Cervus nippon*) and red deer (*Cervus elaphus*) in Germany. Pathogens.

[71] Tegtmeyer P., Ganter M., von Loewenich F.D. (2019). Simultaneous infection of cattle with different *Anaplasma phagocytophilum* variants. Ticks Tick-borne Dis..

[72] Overzier E., Pfister K., Thiel C., Herb I., Mahling M., Silaghi C. (2012). *Anaplasma phagocytophilum* in questing *Ixodes ricinus* ticks: Comparison of prevalences and partial 16S rRNA gene variants in urban, pasture, and natural habitats. Appl. Environ. Microbiol..

[73] Schorn S., Pfister K., Reulen H., Mahling M., Manitz J., Thiel C., Silaghi C. (2011). Prevalence of *Anaplasma phagocytophilum* in *Ixodes ricinus* in Bavarian public parks, Germany. Ticks Tick-borne Dis..

[74] Silaghi C., Kohn B., Chirek A., Thiel C., Nolte I., Liebisch G., Pfister K. (2011). Relationship of molecular and clinical findings on *Anaplasma phagocytophilum* involved in natural infections of dogs. J. Clin. Microbiol..

[75] Adamska M. (2020). The role of different species of wild ungulates and *Ixodes ricinus* ticks in the circulation of genetic variants of *Anaplasma phagocytophilum* in a forest biotope in north-western Poland. Ticks Tick-borne Dis..

[76] Jahfari S., Coipan E.C., Fonville M., van Leeuwen A.D., Hengeveld P., Heylen D., Heyman P., van Maanen C., Butler C.M., Földvári G. (2014). Circulation of four *Anaplasma phagocytophilum* ecotypes in Europe. Parasites Vectors.

[77] Petrovec M., Sumner J.W., Nicholson W.L., Childs J.E., Strle F., Barlicč J., Lotricč-Furlan S., Županc T.A. (1999). Identity of ehrlichial DNA sequences derived from *Ixodes ricinus* ticks with those obtained from patients with human granulocytic ehrlichiosis in Slovenia. J. Clin. Microbiol..

[78] Liz J.S., Sumner J.W., Pfister K., Brossard M. (2002). PCR detection and serological evidence of granulocytic ehrlichial infection in roe deer (*Capreolus capreolus*) and chamois (*Rupicapra rupicapra*). J. Clin. Microbiol..

[79] Petrovec M., Bidovec A., Sumner J.W., Nicholson W.L., Childs J.E., Avsic-Zupanc T. (2002). Infection with *Anaplasma phagocytophila* in cervids from Slovenia: Evidence of two genotypic lineages. Wien. Klin. Wochenschr..

[80] Rar V.A., Epikhina T.I., Livanova N.N., Panov V.V., Doroschenko E.K., Pukhovskaya N.M., Vysochina N.P., Ivanov L.I. (2011). Genetic variability of *Anaplasma phagocytophilum* in *Ixodes persulcatus* ticks and small mammals in the Asian part of Russia. Vector Borne Zoonotic Dis..

[81] de la Fuente J., Bussche R.A.V.D., Kocan K.M. (2001). Molecular phylogeny and biogeography of North American isolates of *Anaplasma marginale* (Rickettsiaceae: Ehrlichieae). Vet. Parasitol..

[82] Chartier C., Paraud C. (2012). Coccidiosis due to *Eimeria* in sheep and goats, a review. Small Rumin. Res..

[83] Sargison N. (2008). Sheep Flock Health: A Planned Approach.

[84] Sargison N., Scott P. (2010). The implementation and value of diagnostic procedures in sheep health management. Small Rumin. Res..

[85] West D.M., Bruère A.N., Ridler A.L. (2018). The Sheep—Health, Disease and Production.

[86] Bath G.F., van Wyk J.A. (2009). The Five Point Check© for targeted selective treatment of internal parasites in small ruminants. Small Rumin. Res..

[87] Piaton E., Fabre M., Goubin-Versini I., Bretz-Grenier M., Courtade-Saïdi M., Vincent S., Belleannée G., Thivolet F., Boutonnat J., Debaque H. (2016). Guidelines for May-Grünwald-Giemsa staining in haematology and non-gynaecological cytopathology: Recommendations of the French Society of Clinical Cytology (SFCC) and of the French Association for Quality Assurance in Anatomic and Cytologic Pathology (AFAQAP). Cytopathology.

[88] Lepherd M., Canfield P., Hunt G., Bosward K. (2009). Haematological, biochemical and selected acute phase protein reference intervals for weaned female Merino lambs. Aust. Vet. J..

[89] Ganter M., Bostedt H., Ganter M., Hiepe T. (2019). Referenzwerte. Klinik der Schaf-und Ziegenkrankheiten.

[90] World Health Organization (2011). Haemoglobin Concentrations for the Diagnosis of Anaemia and Assessment of Severity.

[91] Humann-Ziehank E., Ganter M., Michalke B. (2016). Selenium speciation in paired serum and cerebrospinal fluid samples of sheep. J. Trace Elements Med. Biol..

[92] Puls R. (1994). Mineral Levels in Animal Health: Diagnostic Data.

[93] Eckert J., Braun R., Shirley M.W., Coudert P. (1995). Guidelines on Techniques in Coccidiosis Research.

[94] Estrada-Peña A., Bouattour A., Camicas J.-L., Walker A.R. (2004). Ticks of Domestic Animals in the Mediterranean Region: A Guide to Identification of Species.

[95] Stuen S., Bergström K. (2001). Serological investigation of granulocytic *Ehrlichia* infection in sheep in Norway. Acta Vet. Scand..

[96] Courtney J.W., Kostelnik L.M., Zeidner N.S., Massung R.F. (2004). Multiplex real-time PCR for detection of *Anaplasma phagocytophilum* and *Borrelia burgdorferi*. J. Clin. Microbiol..

[97] Michelet L., Delannoy S., Devillers E., Umhang G., Aspan A., Juremalm M., Chirico J., van der Wal F.J., Sprong H., Boye Pihl T.P. (2014). High-throughput screening of tick-borne pathogens in Europe. Front. Cell. Infect. Microbiol..

[98] Yousefi A. (2018). Phylogenetic analysis of *Anaplasma marginale* and *Anaplasma ovis* isolated from small ruminant based on MSP4 gene in western regions of Iran. Comp. Clin. Pathol..

[99] Massung R.F., Slater K., Owens J.H., Nicholson W.L., Mather T.N., Solberg V.B., Olson J.G. (1998). Nested PCR assay for detection of granulocytic ehrlichiae. J. Clin. Microbiol..

[100] de la Fuente J., Massung R.F., Wong S.J., Chu F.K., Lutz H., Meli M., von Loewenich F.D., Grzeszczuk A., Torina A., Caracappa S. (2005). Sequence analysis of the msp4 gene of *Anaplasma phagocytophilum* strains. J. Clin. Microbiol..

[101] Alberti A., Zobba R., Chessa B., Addis M.F., Sparagano O., Pinna Parpaglia M.L., Cubeddu T., Pintori G., Pittau M. (2005). Equine and canine *Anaplasma phagocytophilum* strains isolated on the island of Sardinia (Italy) are phylogenetically related to pathogenic strains from the United States. Appl. Environ. Microbiol..

