# Supplementary material for: Anaplasma phagocytophilum and Anaplasma ovis–Emerging Pathogens in the German Sheep Population
Source: Pathogens. 2021 Oct 9;10(10):1298. doi: 10.3390/pathogens10101298 (PMC8537415; doi:10.3390/pathogens10101298)
Supplement: Supplementary file 1 [file pathogens-10-01298-s001.zip › Supplementary Figures 1-3.pdf]

Anaplasma phagocytophilum **and** *Anaplasma ovis* **– emerging pathogens in the German sheep population**

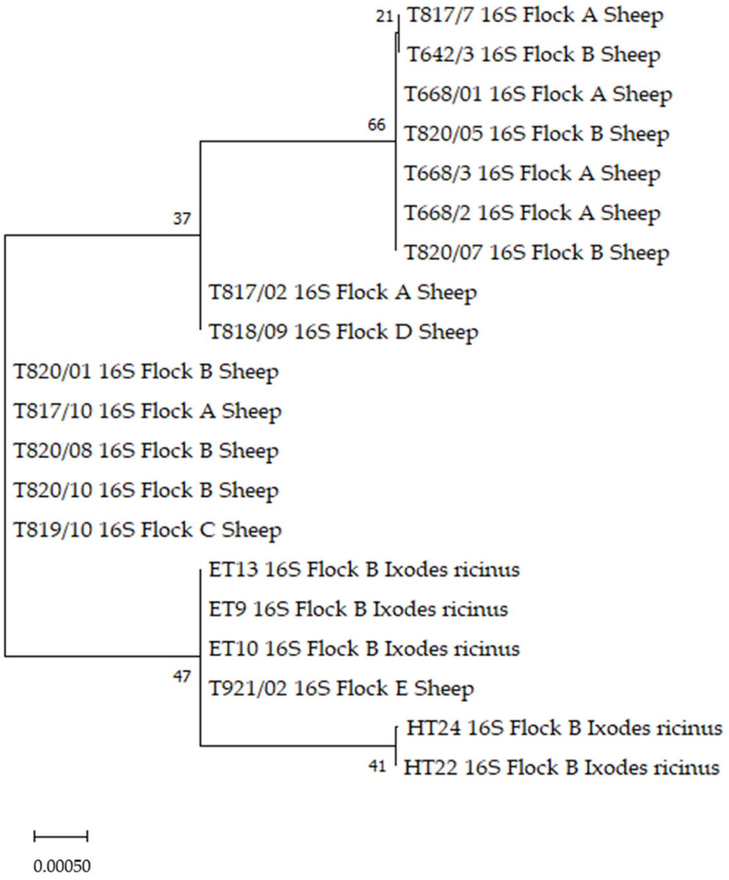

Figure S1. Phylogenetic tree of *Anaplasma phagocytophilum 16S rRNA* sequences derived from sheep and *Ixodes ricinus* ticks. The evolutionary analyses were conducted with MEGA X [40] using the Maximum Likelihood method and Jukes-Cantor model [Jukes, T.H.; Cantor, C.R. Evolution of protein molecules. In *Mammalian Protein Metabolism*; Munro, H.N., Ed.; Academic Press: New York, NY, USA, 1969; pp. 21–132]. Statistical support was calculated by 1000 bootstrap replicates, and the tree is scaled with branch lengths indicating the number of substitutions per site.

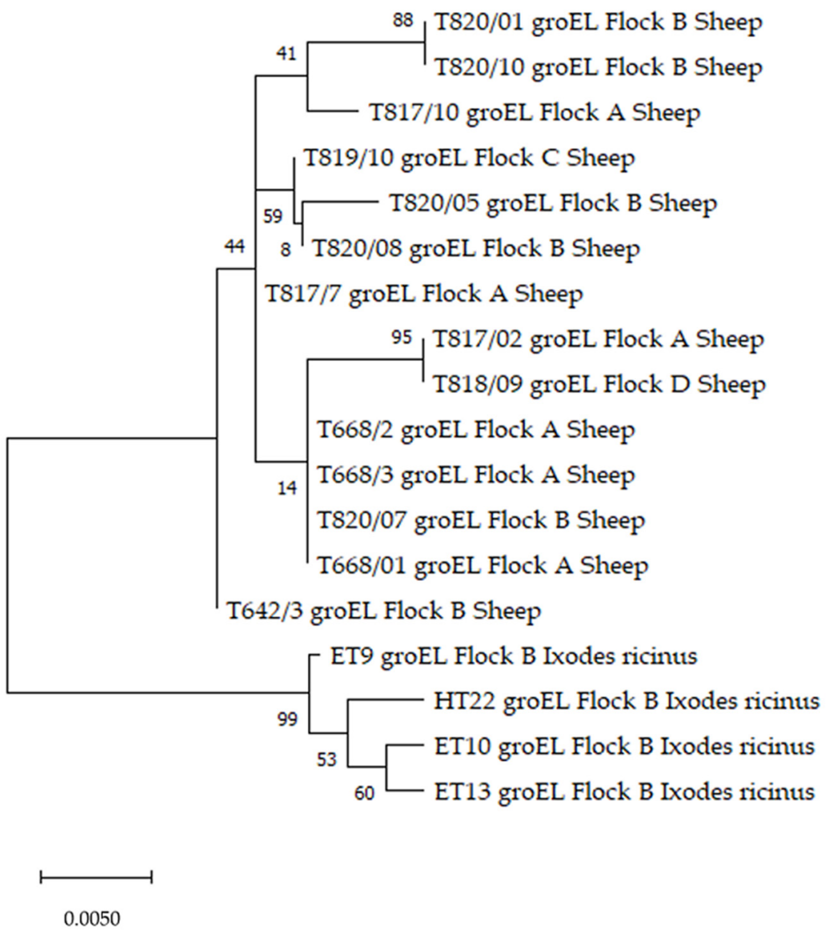

Figure S2. Phylogenetic tree of *Anaplasma phagocytophilum groEL* sequences derived from sheep and *Ixodes ricinus*. The evolutionary analyses were done with MEGA X [40] using the Maximum Likelihood method and Tamura 3-parameter model [41]. Statistical support was calculated by 1000 bootstrap replicates, and the tree is scaled with branch lengths indicating the number of substitutions per site.

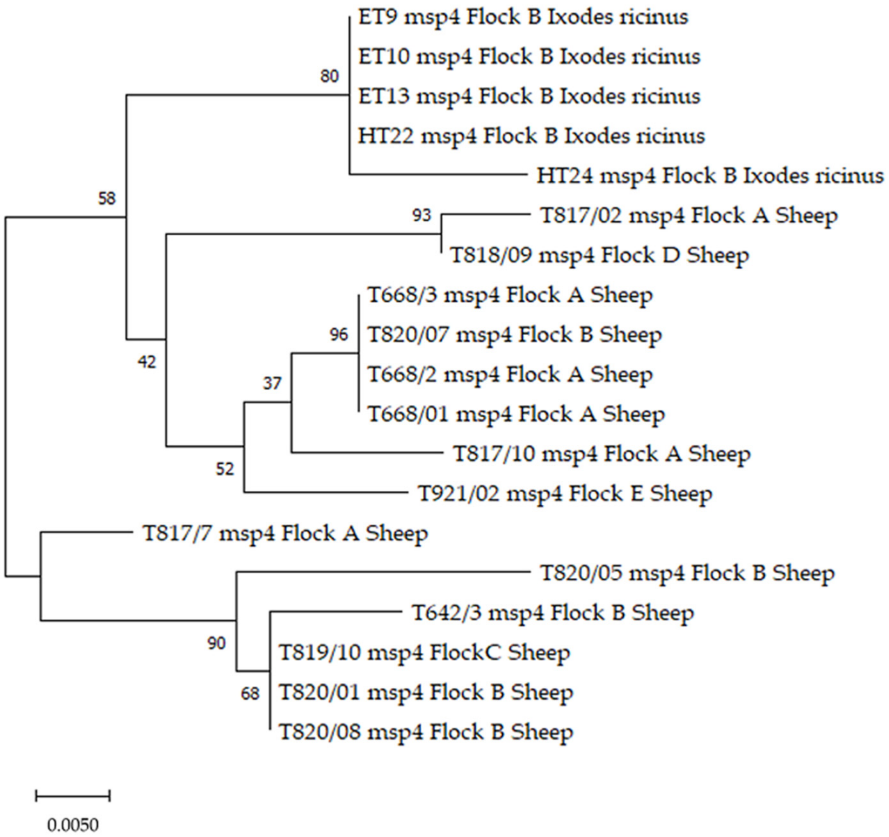

Figure S3. Phylogenetic tree of *Anaplasma phagocytophilum msp4* sequences from sheep and *Ixodes ricinus*. The evolutionary analyses were conducted in MEGA X [40] using the Maximum Likelihood method and Tamura 3-parameter model using a discrete Gamma distribution [41]. Statistical support was calculated by 1000 bootstrap replicates, and the tree is scaled with branch lengths indicating the number of substitutions per site.
