# Supplementary material for: Anaplasma phagocytophilum and Anaplasma ovis–Emerging Pathogens in the German Sheep Population
Source: Pathogens. 2021 Oct 9;10(10):1298. doi: 10.3390/pathogens10101298 (PMC8537415; doi:10.3390/pathogens10101298)
Supplement: Supplementary file 1 [file pathogens-10-01298-s001.zip › Supplementary Table 3.pdf]

***Anaplasma phagocytophilum* and *Anaplasma ovis* – emerging pathogens in the German sheep population**

Table S3. BLAST results retrieved for *Anaplasma phagocytophilum 16S rRNA*, *groEl* and *msp4* sequences.

Flock	Sample ID	*16S rRNA*		*groEL*		*msp4*	
		GenBank Access. No.	Identity (%)	GenBank Access. No.	Identity (%)	GenBank Access. No.	Identity (%)
A	T668.1 ewe	MK814406	99.8	KR092132	99.5	KM205425	100
	T668.2 lamb		100		99.8		100
	T668.3 lamb		100		99.8		100
	T817.2 lamb	MN658588	100	KF031399	100	MF974858	100
	T817.3 lamb	n/a	n/a	n/a	n/a	n/a	n/a
	T817.7 lamb	MK814406	99.6	KJ832471	99.7	KM205443	100
	T817.10 lamb	KP276588	100	HM057224	99.5	MF974854	99.5
B	T642.1 lamb	n/a	n/a	n/a	n/a	n/a	n/a
	T642.3 lamb	MK814406	99.5	KF312359	99.5	KM205427	99.5
	T820.1 lamb	KP276588	99.8	MK341070	98.6	KM205420	100
	T820.5 lamb	MK814406	99.8	MG670108	99.5	MF974848	98.1
	T820.7 lamb		100	KR092132	99.7	KM205425	100
	T820.8 lamb	KP276588	100	MG670108	99.7	KM205420	100
	T820.10 lamb		100	MK341070	99.4	n/a	n/a
C	T819.10 lamb		100	MG670108	99.8	KM205420	100
D	T818.9 lamb	MK814412	100	KF031399	99.8	MF974858	100
E	T921.2 lamb	JN181070	99.8	n/a	n/a	KM205425	99.2
B	ET-9 tick		100	AY281848	99.8	KM205439	100
	ET-10 tick		100	KM215256	99.8		100
	ET-13 tick		100	KF312356	99.7		100
	HT-22 tick	KC800983	100	HQ629904	99.8		100
	HT-24 tick		100	n/a	n/a	EF067342	99.7
